# Trajectories of psychological distress for Australian fathers parenting a child on the autism spectrum: Evidence from early childhood to adolescence

**DOI:** 10.1177/13623613241272005

**Published:** 2024-09-05

**Authors:** Monique Seymour, Laura Pecora, Grace McMahon, Catherine E Wood, Mark Feinberg, Rob Hock, Rebecca Giallo

**Affiliations:** 1 Deakin University, Australia; 2 Murdoch Children’s Research Institute, Australia; 3 The Royal Children’s Hospital, Australia; 4 Swinburne University of Technology, Australia; 5 The Pennsylvania State University, USA; 6 University of South Carolina, USA

**Keywords:** autism, father, mental health, psychological distress, trajectory

## Abstract

Most research on the mental health of fathers with children on the autism spectrum has been cross-sectional. Little is currently known about the trajectories of fathers’ mental health across their parenting journey. Using longitudinal latent class analysis, this study aimed to: (1) report on the estimates of fathers’ psychological distress across six timepoints, from when their children were aged 4–14 years; (2) identify classes of fathers as defined by their trajectory of distress over time; and (3) identify early psychosocial risk factors associated with the identified trajectories of psychological distress. Participants were 281 fathers of children on the autism spectrum who participated in the Longitudinal Study of Australian Children. Longitudinal latent profile analysis across the six timepoints of data identified a two-class model best fit the data, with profiles reflecting minimal distress (*n* = 236, 84%) and elevated and increasing distress (*n* = 45, 16%). Regression analysis revealed ongoing medical conditions and heightened interparental conflict as significant predictors to sustained psychological distress. Findings highlight that psychological distress persists for a substantial portion of fathers throughout their child’s development. Strengthening fathers’ physical health and enhancing the couple relationship are potential avenues for positively impacting fathers’ long-term mental health.

**Lay Abstract:**

This study explores the mental health journey of fathers with children on the autism spectrum. Little is known about mental health over time for these fathers. This research spans six-timepoints from when children were aged 4 to 14 years, to track fathers’ mental health. This study had three aims: (1) report estimates of fathers’ psychological distress across 10 years of child development; (2) identify separate courses of psychological distress over time; and (3) identify early risk factors associated with these courses. This study used data from 281 fathers of children on the autism spectrum who took part in the Longitudinal Study of Australian Children. Using a statistical method to group fathers based on their psychological distress scores over 10 years of child development, the results showed that two groups best explained the data; this included a group of fathers who experienced low levels of psychological distress over the 10 years of child development (84%), and another group of fathers who experienced heightened psychological distress across this time (16%). Further analysis showed that fathers who had an ongoing medical condition and higher levels of interparental conflict with their partners were more likely to be in the heightened psychological distress group. These findings show that almost one in six fathers deal with persistent psychological distress throughout their child’s early childhood and into early adolescence. This study advocates for interventions focusing on improving fathers’ physical health and the couple relationship as ways to positively impact fathers’ mental health in the long run.

## Introduction

Fathers are gaining increasing recognition for their importance within families due to increased child caregiving contributions over recent decades. While fatherhood has been associated with increased happiness and well-being for many (Nelson-Coffey et al., 2019) it also places some men at an increased risk of experiencing mental health and well-being difficulties such as psychological distress (i.e. symptoms of depression, anxiety, and stress; Kotelchuck, 2022). Due to increased parenting demands and higher levels of family stress, fathers of children on the autism spectrum are at an increased risk of experiencing mental health difficulties than fathers in the general population (Hartley et al., 2012; Hastings & Brown, 2002; Seymour et al., 2017). Within Australia, a population-based study indicated that 17% of fathers of 8- to 9-year-old autistic children experienced elevated psychological distress (Seymour et al., 2017). This is consistent with estimates from other studies of fathers from high income countries. For example, in a UK study of 20 fathers of primary school-aged children (*M* = 12 years), 15% reported clinically significant depressive symptoms and 25% reported clinically significant anxiety symptoms (Hastings & Brown, 2002). In two US-based studies, almost a third of fathers of autistic adolescents (*M* = 16 years) reported clinically significant depressive symptoms (Hartley et al., 2012), and 8% of fathers of school-aged autistic children (*M* = 9 years) had an existing formal diagnosis of depression (Cohrs & Leslie, 2017). Mental health difficulties not only have direct impacts on fathers’ health and well-being, but can affect family functioning (Sell et al., 2021), partner mental health (Thiel et al., 2020) and children’s social-emotional outcomes (Dachew et al., 2023). While these studies highlight the increased risks of mental health difficulties for fathers of children on the autism spectrum, the vast majority of this research has been cross-sectional. Little is currently known about the course of fathers’ mental health across their parenting journey.

The demands of parenting change as children grow and develop (Frosch et al., 2021). A recent longitudinal study found that over a 10-year period (4–14 years) behavioural problems for autistic children peak at 10 years of age (May & Williams, 2024). At the same time, psychological distress and negative affect also increase with child age for these mothers (May & Williams, 2024). Yet no known study has investigated whether mental health difficulties persist across the childhood-adolescence period for fathers of children on the autism spectrum. Not all fathers are likely to experience mental health difficulties, therefore understanding the trajectories of fathers’ mental health difficulties when parenting a child on the autism spectrum is crucial in providing targeted and appropriate support at key parenting stages.

Identifying psychosocial factors associated with mental health difficulties for fathers, especially enduring mental health difficulties, is needed to inform service providers of which fathers may be at greatest risk. Previous cross-sectional research identified a range of social determinants of mental health for 159 fathers of 8- to 9-year-old autistic children (Seymour et al., 2018). These included individual characteristics (i.e. experiencing depression within the past year, parenting self-efficacy), interpersonal characteristics (i.e. partner mental health, relationship quality, and social support) and social environmental factors (i.e. experiencing financial hardship and poor job quality). In parents of autistic children, child sex and coexisting conditions (e.g. health/medical conditions and behavioural difficulties) have also been found to influence parent mental health (May & Williams, 2024; Zamora et al., 2014). For example, parents of female autistic children (aged 1–15 years) experience higher stress than parents of male autistic children (Zamora et al., 2014). Similarly, research relating to fathers of children with and without disabilities has found that a range of individual (e.g. paternal age and medical conditions), interpersonal (e.g. relationship difficulties, child behaviour, child sleep difficulties, partner mental health) and social environment (e.g. stressful life events, employment, education, immigration, remoteness) factors are associated with fathers’ experiences of psychological distress (Ahmad & Dardas, 2015; Dunn et al., 2001; Falk et al., 2014; Giallo et al., 2015; Goodman, 2004; Hartley et al., 2012; Hastings & Brown, 2002; Hastings et al., 2005; Kayfitz et al., 2010; Nath et al., 2016; Olsson & Hwang, 2008; Ramchandani et al., 2008). This research is largely cross-sectional and not all fathers will follow the same trajectory of mental health (Giallo et al., 2014; Nieh et al., 2022). To build on this research, it is crucial to explore the psychosocial factors that place fathers at increased risk of mental health difficulties over time.

Determining the extent to which fathers of autistic children experience mental health difficulties over early childhood to adolescence is important for informing the ideal timing of mental health interventions. Identifying risk factors for sustained mental health difficulties can aid early identification and targeted support. This has direct benefits to fathers, children (Giallo et al., 2013; Hastings & Brown, 2002; Kamis, 2021), partners and families (Jellett et al., 2015; Langley et al., 2017), along with broader policy and economic benefits to the Australian healthcare system. Therefore, this study sought to examine the mental health of fathers of children on the autism spectrum over a 10-year period drawing data from a large population-based study of Australian children and their families. Specifically, the aims study were to (1) report on the estimates of fathers’ psychological distress across six timepoints when their children were aged 4–5, 6–7, 8–9, 10–11, 12–13 and 14–15 years; (2) identify subgroups of fathers as defined by their trajectory of distress over time; and (3) identify early psychosocial risk factors associated with the identified trajectories of psychological distress. Hypotheses were not formulated due to the exploratory nature of the research aims.

## Methods

### Procedure and participants

Secondary data analysis from the Growing Up in Australia: Longitudinal Study of Australian Children (LSAC) was used in this study. Ethics approval for LSAC was granted by the Australian Institute of Family Studies. Comprehensive details about the study design, sample frame and procedures, and data collection methods are published elsewhere (Soloff et al., 2005). Briefly, there are two cohorts: (1) the Baby cohort (B-cohort) when children were recruited in the first year of life, and (2) the Kindergarten cohort (K-cohort) when children were recruited at age 4–5 years. For both cohorts, a two-staged clustered sample design was used. Approximately 10% of all Australian postcodes which were stratified by state of residence and urban versus rural status were initially selected. Next, a number of children proportional to population size were randomly selected from each postcode using Australia’s universal health insurance database (Medicare). The study began in 2004 and has completed nine biennial follow-ups. At Wave 1, the B-cohort consisted of 5107 children aged 3–12 months and the K-cohort consisted of 4983 children aged 4–5 years. By consequence, the age of the children in the two cohorts began to overlap from Wave 3.

Data for this study are drawn from the B- and K-cohorts when child ages overlap at 4–5 years (B-cohort Wave 3, K-cohort Wave 1), 6–7 years (B-cohort Wave 4, K-cohort Wave 2), 8–9 years (B-cohort Wave 5, K-cohort Wave 3), 10–11 years (B-cohort Wave 6, K-cohort Wave 4), 12–13 years (B-cohort Wave 7, K-cohort Wave 5) and 14–15 years (B-cohort Wave 8, K-cohort Wave 6). Given that preliminary data analysis failed to reveal any significant cohort effects (Australian Institute of Family Studies, 2011), data from the two cohorts were combined. Retention was lower for families of Aboriginal and Torres Strait Islander and non-English speaking backgrounds, parents with low educational attainment, and for families living in rental properties (Mission & Sipthorp, 2007).

There were 381 children on the autism spectrum who were initially identified by parent report (i.e. ‘does study child have any of these ongoing conditions: autism, Asperger’s, or other autism spectrum?’). This information was provided by the primary caregiver (93.4% mothers) at child ages 6–7, 8–9, 10–11, 12–13, and 14–15 years, for both cohorts. Information on autism diagnosis was not collected at child aged 4–5 years. This was autism is known to be a stable diagnosis across childhood (May et al., 2021; Woolfenden et al., 2012), and children who no longer meet criteria tend to still show delays in one or more neurodevelopmental areas (Benedetto et al., 2021). As such, an autism diagnosis was implied at this timepoint as the mean age of diagnosis for the current sample was 48.37 months (4.03 years). While autism diagnosis was not confirmed, parent report of child autism diagnosis and broader neurodiversity has been found to be valid (Cree et al., 2023; Daniels et al., 2012).

From the B-cohort 197 biological, adoptive or step- fathers were identified and 110 fathers from the K-cohort. Twenty-six cases did not have K6 data available for fathers at any timepoint and were removed from the sample. The final analysis sample included 281 fathers of children on the autism spectrum. The demographic characteristics of the study children and their fathers are provided in Table 1. The majority of children on the autism spectrum were male (73.8%). Most fathers were born in Australia, spoke English at home, had an educational attainment of Year 12 or above, were in paid employment and in a couple relationship. When compared with fathers in the analysis sample, fathers who were excluded were more likely to have experienced financial hardship and not be in paid employment (*p* < 0.05).

**Table 1.** Potential predictor variables, as measured at child age 4–5 years.

Construct	Measure (source)	Additional information
*Individual characteristics*		
Age		Age of father at last birthday (years).
Father reported health/medical condition		No = 0; Yes = 1. Concurrent health or medical condition that has lasted or likely to last 6 or more months. Conditions included sight, hearing, and speech problems, learning difficulties, breathing difficulties, chronic pain, mental illness.
*Interpersonal characteristics*		
Child gender		Male = 0; Female = 1.
Child health/ medical condition		No = 0; Yes = 1. As above for fathers’ health/medical condition.
Child behaviour	Total Difficulties of the Strengths and Difficulties Questionnaire (Goodman, 1997)	20 items rated by primary caregiver, for example ‘Restless, overactive, cannot stay still for long’ and ‘Often seemed worried’. Rating scale from 0 = *Not true* to 2 = *Certainly true*; higher scores reflect more emotional or behaviour difficulties for the child. Cronbach’s α = 0.77.
Child sleep problems		No = 0; Yes = 1. Four items rated by the primary caregiver, for example ‘difficulty getting to sleep’ and ‘not happy to sleep alone’. Cronbach’s α = 0.69.
Mothers’ mental health	Kessler-6 (K6; Kessler et al., 2003)	As above for fathers’ psychological distress. Cronbach’s α = 0.83.
Interparental Conflict	Inter-Parental Conflict subscale from the Quality of Co-parental Interactions Scale (Ahrons, 1981).	Four items assessed verbal conflict between parents (e.g., disagreements, arguments, anger, and hostility), and one item assessed physical conflict (e.g., arguments with pushing, hitting, kicking, or shoving). Fathers were asked to indicate how often these behaviours occur on a 5-point scale (1 = *Never* to 5 = *Always*). Items summed; higher scores indicate higher conflict. Cronbach’s α = 0.80.
Number of children		Number of children in home; continuous.
*Social environment characteristics*		
Stressful life events		Number of life stressors (e.g., parent, partner or child death; illness, injury or assault; major financial crisis; legal problems; relationship separation). No stressors = 0; one or more events = 1.
Educational attainment		Completed year 12 or equivalent = 0; did not complete = 1.
Employment status		Part-time/Full-time = 0; not in paid employment = 1
Country of birth		Australia = 0; other = 1.
Income		Yearly income. ⩽US$25,999 = 1; US$26,000- US$51,999 = 2; US$52,000- US$103,999 = 3; ⩾ US$104,000 = 4.
Remoteness		Major city = 0; Regional or remote area = 1.
Financial hardship	Hardship scale (Bray, 2001)	Six items assessing whether family went without meals, was unable to heat/cool home, etc. in last 12 months. Higher scores indicate family experiencing greater financial hardship. Cronbach’s α = 0.58.
Job quality	Job Quality Index for Parents (Strazdins et al., 2010)	Five items assessing access to paid parental leave, flexible hours, job control, job security. Coded so that higher scores indicate positive employment conditions, then dichotomised so that 0 = one or more favourable conditions; 1 = no favourable conditions. Cronbach’s α = 0.38.

### Measures

*Psychological distress* was measured using the Kessler-6 (K6; Kessler et al., 2003). Fathers reported on the extent to which they felt nervous, hopeless, restless, extremely sad, and worthless in the last 4 weeks. The six items were rated on a 5-point scale, ranging from 0 = *None of the time* to 4 = *All or most of the time*. All items were summed, with higher scores indicating greater psychological distress. The K6 has strong psychometric properties and is often used to screen for serious mood and anxiety disorders (Kessler et al., 2002). To describe the severity and proportion of psychological distress at each timepoint, consistent with other studies using LSAC data (Giallo et al., 2014; Westrupp et al., 2015), a cut point of eight or more was used to indicate symptomatic or clinically significant psychological distress. To explore trajectories of psychological distress, the continuous total score was used. Internal consistency for the current sample, as measured by Cronbach’s α, ranged from 0.81 when children were aged 6–7 years to 0.88 when children were aged 12–13 years.

#### Potential predictor variables

Research has shown that several factors may be associated with psychological distress experienced by fathers in the general community (Giallo et al., 2014, 2015) and fathers of autistic children (Seymour et al., 2017). Guided by an ecological perspective (Bronfenbrenner & Morris, 2006; Rogers et al., 2009) and previous research, potential predictor variables at child age 4–5 years were explored (Table 2). Predictors are classified within individual, interpersonal and social-environmental domains. As summarised in the table, LSAC employed standardised measures from national studies where possible.

**Table 2.** Child and father demographic characteristics at child age of 4–5 years.

Variable	*n* (%) Autistic children (*n* = 381)
Gender, male	281 (73.8%)
Missing	13 (3.4%)
Age (years), *M* (*SD*)	4.18 (0.39)
Age of autism diagnosis (months), *M* (*SD*)^a^	48.37 (19.57)
Primary caregiver gender female	356 (93.4%)
Missing	13 (3.4%)
	Fathers (*n* = 281)
Age (years), *M* (*SD*)	37.32 (5.96)
Australia born	236 (84%)
Aboriginal or Torres Strait Islander	3 (1.1%)
English as main language	263 (93.6%)
Financial hardship, *M* (*SD*)	0.39 (0.81)
Education	
Year 12 or above	165 (58.7%)
Employment status	
Full-time/part-time	273 (97.2%)
Not in paid employment	8 (2.8%)
Father relationship to study child	
Biological	274 (97.5%)
Adoptive	1 (0.4%)
Step-parent	6 (2.1%)
Couple parent family	278 (98.9%)
Two or more children in household	247 (87.9%)

a As reported at 6/7 years of age.

#### Data analysis

Descriptive statistics for sample demographics and psychological distress at each timepoint (Aim 1) were conducted in SPSS version 29 (IBM Corp, 2021). Next, longitudinal latent profile analysis (LLPA) was conducted to identify distinct trajectories (Aim 2) of fathers’ psychological distress over six timepoints (when the study children were aged 4–5 years, 6–7 years, 8–9 years, 10–11 years, 12–13 years and 14–15 years) using MPlus version 8.7 (Muthén & Muthén, 2023). This approach involved beginning with a one-class model and subsequently fitting models with increasing numbers of classes to identify the smallest number of classes that best fit the associations in the data. Given this is a data-driven approach, a range of criteria were considered when determining the best fitting model (Berlin et al., 2014). When comparing models, lower values for the likelihood ration statistic (L2), Akaike information criterion (AIC), and the Bayesian information criterion (BIC) indicated better fitting models. The Entropy value was also considered to determine the accuracy with which the model’s classified fathers into their most likely class, with values >0.80 indicating good classification accuracy. The Vuong–Lo–Mendell–Rubin likelihood ratio test was used to examine the improvement between neighbouring class models (i.e. comparison of two vs three classes), with values <0.05 indicating a statistically significant improvement in model fit when an additional class is added. Finally, the clinical meaningfulness and class sizes were considered when making a final decision about model solutions. After determining the final model solution, the class membership of fathers for psychological distress was saved and used in subsequent analyses conducted in SPSS.

Finally, a multivariate logistic regression was performed in SPSS to identify psychosocial risk factors associated with trajectories of psychological distress (Aim 3), with minimal distress as the reference group. Correlations were conducted to assess the bivariate relationships between the latent class profiles and predictor variables. Only predictors that were significantly (*p* < 0.05) correlated with fathers’ psychological distress at least one timepoint (see Supplementary Table) were included in the final multivariate model. The most common response category was coded as the reference group for binary variables (e.g. Aboriginal or Torres Strait Islander: 0 = No (ref) 1 = Yes). All variables of interest were entered simultaneously. Results are presented as odds ratios with 95% confidence intervals.

For Aim 2, missing data were handled using full information maximum likelihood (FIML) in MPlus. FIML is a commonly used missing data technique which uses all available data for individual cases to estimate model parameters and requires cases to have data for at least one variable of interest in the model to be estimated. For Aim 3, missing data were handled using multiple imputation (MI) in SPSS. The results for the regression analysis were pooled across 20 parallel imputed datasets incorporating variables that influence missing responses (i.e. number of children in household and financial hardship) along with all analysis variables.

### Community involvement

Community members were not involved in any aspect of the research process.

## Results

### Data screening and descriptives

After excluding the 26 cases outlined above, missing data ranged from 0.4% (on study child medical condition) to 22.1% on fathers’ K6. Data were not missing at random, Little’s Missing Completely At Random χ^2^ = 308.21, *df* = 150, *p* < 0.001. As the pattern of results for cases with complete data and those with imputed data was similar, only results for cases with imputed data are reported here (Sterne et al., 2009).

The descriptives and estimates for psychological distress (K6) at each timepoint are presented in Table 3 (Aim 1). For the overall sample, the mean scores for psychological distress were generally stable at each timepoint, while the proportion of fathers at the symptomatic cut point was highest when their child was aged 4–5 years, followed by 8–9 years and then 14–15 years. Statistical and graphical measures of normality revealed that distributions for psychological distress at each wave were generally positively skewed. Therefore, robust maximum likelihood estimation was used to adjust the fit indices and parameter estimates to account for non-normality when conducting the LLPA. Prior to conducting the regression analysis, bivariate correlations among all study variable and potential predictor variables were conducted. Only variables with significant zero-order correlations with psychological distress for fathers of autistic children were entered into the regression model.

**Table 3.** Descriptive statistics for psychological distress as measured by the K6.

	Range	*M* (*SD*)	Proportion of fathers above the “symptomatic” cut-point, *n* (%)	Skewness	Kurtosis
Child aged 4–5 years	0–24	3.88 (3.60)	33 (11.7)	1.51	2.87
Child aged 6–7 years	0–24	3.60 (3.31)	25 (8.9)	1.30	1.72
Child aged 8–9 years	0–24	3.55 (3.78)	29 (10.3)	1.61	3.12
Child aged 10–11 years	0–24	3.11 (3.26)	19 (6.8)	1.54	2.86
Child aged 12–13 years	0–24	3.61 (3.91)	23 (8.2)	1.57	2.73
Child aged 14–15 years	0–24	3.93 (3.96)	26 (9.2)	1.57	2.73

### Trajectories of fathers’ psychological distress

To identify subgroups of fathers as defined by their trajectory of psychological distress over time (Aim 2), LLPA was conducted. Models were estimated for one to four classes (see Table 4). After inspection of all model fit indexes, the two-class model was selected as the final model. The L2, AIC, and BIC for the two-class model were lower than the one-class model, suggesting improved model fit with the addition of a second class. The two-class model had the highest entropy and high average posterior probabilities (class one = 0.99, class two = 0.96), indicating good accuracy in assigning fathers to their most likely class. The Vuong–Lo–Mendell–Rubin statistic indicated a significant difference between the 1- and 2-class models, suggesting that the 2-class model gives significant improvements in fit over the 1-class model. The Vuong–Lo–Mendell–Rubin statistics was not statistically significant for the three- or four-class models.

**Table 4.** Model fit indexes for latent classes of psychological distress.

Model	*L* ^2^	BIC	AIC	Entropy	Vuong–Lo–Mendell–Rubin	*p*-value
1-class	-3003.623	6074.906	6031.246	-	-	-
**2-class**	**-2777.466**	**5662.061**	**5592.933**	**0.921**	**1 vs 2 classes**	**0.0018**
3-class	-2717.818	5582.233	5487.635	0.850	2 vs 3 classes	0.2028
4-class	-2678.128	5542.321	5422.256	0.812	3 vs 4 classes	0.6498

**Figure 1.**
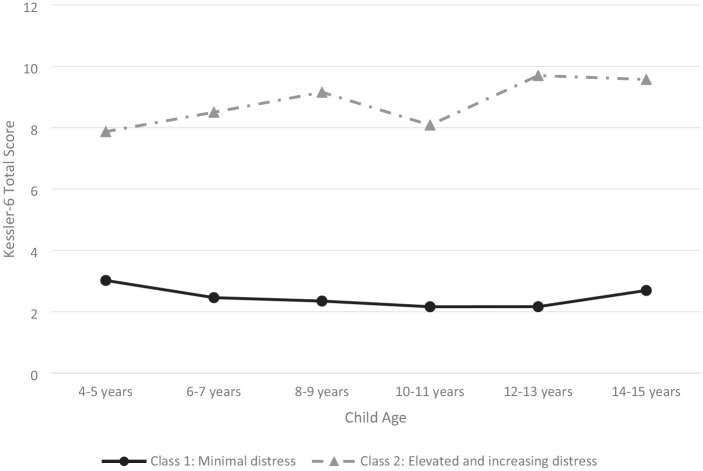
Trajectories of fathers’ psychological distress across childhood and early adolescence.

The trajectories of fathers’ psychological distress over time for the two-class solution are presented in Figure 1. Most fathers were assigned to the trajectory representing a pattern of ‘minimal distress’ (*n* = 236, 84%). This trajectory was characterised by symptoms that were consistently low across all timepoints. A small proportion of fathers (*n* = 45, 16%) were assigned to a trajectory representing a pattern of ‘elevated and increasing’ psychological distress over time. The estimated mean K6 scores for this class were just below the clinical cut-off of 8 at 4–5 years and then increased over time to remain consistently at or above the cut-off across all other timepoints.

Table 5 shows that the majority of fathers within the minimal distress class did not report psychological distress within the symptomatic range (⩾8) on the K6 at any timepoint over the 10 years of child development, while 10.6% of fathers reported elevated psychological distress at one or two timepoints. For fathers within the elevated and increasing class, most fathers reported symptomatic psychological distress at three or more timepoints, while only 2% never experienced symptomatic psychological distress.

**Table 5.** Proportion of fathers reporting elevated psychological distress symptoms (K6 >= 8) over time for each profile.

K6 above the “symptomatic” cut-point	Minimal distress *n* = 236 *n*, (%)	Elevated and increasing distress *n* = 45 *n*, (%)	Total sample *N* = 281
K6 within normal range	211 (89.4)	1 (2.2)	238 (77.5)
Elevated K6 at one timepoint	20 (8.5)	10 (22.2)	30 (9.8)
Elevated K6 at two timepoints	5 (2.1)	6 (13.3)	11 (3.6)
Elevated K6 at three timepoints	0	17 (37.8)	17 (5.5)
Elevated K6 at four timepoints	0	6 (13.3)	6 (2.0)
Elevated K6 at five timepoints	0	2 (4.4)	2 (0.7)
Elevated K6 at six timepoints	0	3 (6.7)	3 (1.0)

### Predictors of latent profile reflecting ‘persistent and increasing distress’

With Class 1: minimal distress as the reference group, the final multivariate model (Aim 3; see Table 6) revealed that the strongest predictors of ‘elevated and increasing distress’ were fathers having an ongoing health/medical condition and fathers’ reports of interparental conflict. Having a greater number of children in the family was approaching significance (*p* = 0.066).

**Table 6.** Predictors of the ‘persistent and increasing psychological distress’ latent profile.

	Class 1: Minimal distress *n* = 236 *n* (%)	Class 2: Elevated and increasing distress *n* = 45 *n* (%)	Final multivariate *n* = 281 OR (95% CI), *p*
Father has ongoing health/medical condition	30.3 (12.8%)	14.0 (31.1%)	**3.40 (1.55**–**7.76), *p* = 0.002**
Child SDQ^a^	35.0	35.8	1.03 (0.95–1.12), *p* = 0.522
Mother K6^a^	4.2	4.5	0.99 (0.90–1.09), *p* = 0.808
Father IPC^a^	9.8	11.1	**1.18 (1.03**–**1.35), *p* = 0.018**
Number of children^a^	2.4	2.6	1.39 (0.98–1.98), *p* = 0.066
Father Aboriginal or Torres Strait Islander	2 (0.01%)	1 (0.2%)	1.30 (0.09–19.02), *p* = 0.849
Father job quality^a^	2.4	2.5	1.13 (0.85–1.51), *p* = 0.406

a Mean; *SD* not produced for MI.

## Discussion

This is the first known population-based study to explore trajectories of psychological distress for Australian fathers of autistic children from early childhood (4–5 years) through to early adolescence (14–15 years). Drawing data from multiple timepoints, two distinct trajectories or patterns of psychological distress over time were identified. Most fathers (84%) were assigned to a class characterised by minimal psychological distress across all timepoints. The majority of fathers in this class did not report elevated psychological distress at any timepoint and approximately 11% reporting elevated symptoms at one to two timepoints. Approximately 16% of fathers were assigned to a class characterised by ongoing elevated psychological distress, which persisted from early childhood to early adolescence. Within this group, 98% of fathers reported elevated psychological distress symptoms at some timepoint over 10 years of child development. While these findings suggested that most fathers of autistic children adjust and cope well over their parenting journey, there is a vulnerable group of fathers who report chronic and ongoing mental health difficulties.

For the overall sample and fathers allocated to the minimal distress group, elevated psychological distress was highest when their children were age 4–5 years. While various factors might account for this increase, this is the time that coincides with autism assessment and diagnosis for many Australian children (Bent et al., 2015; Gibbs et al., 2019). Parent-reported experiences of the autism assessment and diagnostic process are characterised by a range of difficult emotions and appraisals (Legg et al., 2023) including stress, grief and shame (Sakai et al., 2019), confusion over best means of supporting their child (Pearson et al., 2020) and feelings of isolation (Makino et al., 2021). Fathers of autistic children reported greater difficulties coping with the initial negative emotions that accompany a child’s diagnosis (Burrell et al., 2017) and require more support to come to terms with demands associated with autism (Camilleri, 2022) as compared to mothers. Yet, there are fewer opportunities for fathers to express their frustrations and feelings of grief (Burrell et al., 2017). It is therefore possible that high estimates of psychological distress observed at the 4- to 5-year timepoint might reflect the challenges experienced by fathers as they adjust and accept their child’s diagnosis.

This study also identified that heightened psychological distress was persistent for a subgroup of fathers (16%). Their symptoms of psychological distress generally increased over time, reaching a peak when their children were 12–13 years of age. Parenting stress can be high for all parents of adolescent children as they navigate through their child’s transition to secondary school, increased need for individuation and autonomy, along with changes to the dynamics and quality of parent–child relationships (Kochanova et al., 2021). The demands of parenting an adolescent are often compounded for parents of autistic children. Common difficulties including adjusting to changes associated with puberty (Rimmington, 2023), sexuality related issues, and increased sensory needs among autistic adolescents present as a series of additional and complex caretaking challenges that increase risks to parental stress proliferation (Raff et al., 2021). Moreover, the increased importance, yet complexity of peer relationships during adolescence (Rimmington, 2023) paired with difficulties developing friendships and emotional regulation differences in autism, often amplify parental concerns for the safety and increased vulnerabilities of their autistic child (Lievore et al., 2024; Raff et al., 2021). During this period, parents also frequently find themselves exploring specialised services and support designed to meet the unique needs of their adolescent (Kuhn et al., 2018; Nuske et al., 2019; Płatos & Pisula, 2019; Shea et al., 2018). The increased rates of psychological distress among this subgroup of fathers with children aged 14–15 years align with broader autism research citing greater uncertainty, anxiety and psychological distress among parents of autistic adolescents (Cheak-Zamora et al., 2017; Raff et al., 2021).

While various interrelated factors contribute to paternal psychological distress, an ongoing medical condition (including, but not limited to, sight, hearing, speech problems and chronic pain) was the strongest predictor of experiencing elevated and persistent psychological distress for fathers in the current sample. Fathers who reported having one or more medical conditions when their children were aged 4–5 years were over three times more likely to experience a pattern of elevated and increasing psychological distress across 10 years of child development, compared to fathers who did not have an ongoing medical condition. The current measure of ongoing medical conditions was broad and captured previous experiences of mental illness as well, which might account for some of the current association with psychological distress. It is well established that a history of mental illness is a robust predictor of future mental illness (Caspi & Moffitt, 2018), and these findings have also been extended to fathers of children on the autism spectrum (Seymour et al., 2017). In addition, some ongoing medical conditions are likely to impact fathers’ ability to care for their children (Seymour et al., 2017) possibly reducing active involvement in caregiving and engagement in early intervention (Li et al., 2022). As Australian fathers often undertake the role of financial provider in families while also increasingly participating in child-rearing activities (Borgkvist et al., 2021), poor physical health might impact their ability to successfully meet competing life demands. Managing a persistent health condition alongside prioritising their child’s needs may hinder fathers from engaging in activities that alleviate parenting stress and promote mental health (e.g. physical, social, self-care and health care utilisation; Brown et al., 2020; Mount & Dillon, 2014).

Notably, the only other factor found to be significantly associated with a trajectory of elevated and persistent psychological distress within the current analysis was fathers’ reports of interparental conflict when their children were aged 4–5 years. While evidence related to conflict in parents of children on the autism spectrum is sparce (Saini et al., 2015), research has consistently indicated that parents of autistic children tend to report lower levels of relationship satisfaction as compared to parents of children without autism (Sim et al., 2016; Wilkes-Gillan & Bourke-Taylor, 2017). The combined stressors of parenting, life challenges and the unique difficulties associated with raising a child on the autism spectrum can strain coparenting relationships, potentially resulting in conflict and negatively affecting the mental health of fathers (Piro-Gambetti et al., 2023).

### Strengths, limitations and future directions

This is the first known study to examine psychological distress among fathers of children on the autism spectrum over a 10-year period from when their children were 4–5 years to 14–15 years. This study extends current cross-sectional research and offers important evidence that there are critical timepoints for fathers’ mental health during their parenting journey. It also emphasises that there is a significant group of fathers who struggle with their mental health over their child’s development.

This study also has several limitations to note. First, there was attrition across timepoints and under-representation of fathers from lower socioeconomic and cultural backgrounds (fathers not in paid employment, fathers experiencing financial difficulties, Aboriginal or Torres Strait Islander fathers), and fathers who were the primary caregiver or single parents. These considerations are likely to impact the Generalisability of findings. Second, inherent with large cohort studies and secondary data analysis, potential bioecological risk and protective factors were limited. For example, social support has been identified as an important protective factor for psychological distress experienced by fathers of children (aged 8–9 years) on the autism spectrum (Seymour et al., 2017). However, a measure of social support was not asked of parents at 4–5 years in the K-cohort and thus could not be explored. Likewise, autistic traits are often elevated in fathers of children on the autism spectrum (Rubenstein & Chawla, 2018) and are an important individual characteristic to consider when exploring determinants of mental health in this population. However, no data were collected on parent neurodivergence. As such, this was not explored in the current study. Third, the measure of ongoing medical conditions was broad and captured a diverse range of physical and mental health conditions. Given the small numbers of participants identifying as having specific medical conditions, all conditions were grouped together for the purpose of this study. Different ongoing mental and physical health/medical conditions are likely to impact fathers’ well-being and functioning in different ways (Kotelchuck, 2022). As such, grouping a broad range of medical conditions together makes it difficult to disentangle the impact of specific medical conditions on fathers’ mental health. Finally, data relied on self-report (e.g. of mental health and interparental conflict) which can be inherent with methodological limitations such as social desirability and cognitive biases. However, it is fathers’ perceptions and subjective experience of mental health, and life’s challenges and experiences that are of importance and facilitates an understanding from fathers’ point of view. In addition, further research is needed comparing mothers and fathers of autistic children. This would allow for similarities and differences in trajectories and risk factors to be identified. Further exploration of these areas will help inform targeted interventions to support parent mental health which also has the potential to protect or enhance child outcomes. Gaining a more comprehensive understanding of mental health and psychological distress in fathers of autistic children can provide much needed evidence for future parent support programmes to promote health outcomes for the whole family.

## Implications and conclusion

The findings of this study have significant implications for research and clinical practice. Most notably, there has been a concentration of research on mothers of autistic children. However, this study underscores the importance of acknowledging and addressing the unique sources of psychological distress experienced by fathers as they adapt to their child’s diagnosis. This study also identified critical periods for mental health support and intervention, and highlighted the importance of identifying optimal strategies for supporting fathers as their children transition into adolescence.

Notably, the early childhood period (around 4–5 years) coinciding with the average age of diagnosis, appears to be a time of particularly high distress for many fathers. This period presents an opportune time for clinicians to engage fathers with mental health support, identify and address underlying medical conditions, and strengthen couple relationships. Integrating these practices into standard assessment and diagnosis is essential. By promoting the physical and mental health of fathers, and nurturing strong couple relationships, there is the potential to enhance or protect fathers’ mental health over the course of their child’s development. Therefore, clinicians should receive training in assessing parents’ mental health and coping mechanisms, understanding relevant risk factors, and gaining knowledge of appropriate referral and support networks for parents when needed.

It is noteworthy that approximately one in six fathers are at risk of experiencing prolonged and heightened psychological distress while caring for their child on the autism spectrum, emphasising the urgency of reinforcing the healthcare system’s support for parents. It is essential for clinicians working with families of children on the autism spectrum to recognise the time constraints and tendency of parents to prioritise their child and family needs over their own well-being (Iadarola et al., 2019). This is especially true for fathers (Camilleri, 2022; Seymour et al., 2017). Encouraging parents, particularly fathers, to prioritise their mental health is crucial. Clinicians should also be aware of the potential impact of physical health and medical conditions on fathers’ mental well-being and actively connect them with appropriate support, which can yield long-term mental health benefits. Family-based interventions focusing on coparenting relationships and conflict resolution should also be widely available, given the impact of couple conflict on fathers’ mental health over time. Coparenting and relationship interventions have demonstrated effectiveness in reducing parental psychological distress in parents from community samples (Feinberg et al., 2010; Giallo et al., 2022) and emerging evidence for parents of children on the autism spectrum (Hock et al., 2022).

In conclusion, this is the first known population-based investigation of the longitudinal course of psychological distress in fathers of autistic children over a 10-year period. It sheds light on a critical area of research that has been historically underrepresented. While previous research has predominantly focused on mothers, this study underscores sources of psychological distress and the ongoing mental health experiences of fathers of children on the autism spectrum. The findings emphasise the need to address fathers’ physical health and strengthen couple relationships to improve fathers’ long-term mental health outcomes. Moreover, this study highlights that some fathers of autistic children may experience significant mental health challenges during their parenting journey. Timely identification of this at-risk group is crucial to help facilitate appropriate interventions and support.

## Supplemental Material

sj-docx-1-aut-10.1177_13623613241272005 – Supplemental material for Trajectories of psychological distress for Australian fathers parenting a child on the autism spectrum: Evidence from early childhood to adolescence Supplemental material, sj-docx-1-aut-10.1177_13623613241272005 for Trajectories of psychological distress for Australian fathers parenting a child on the autism spectrum: Evidence from early childhood to adolescence by Monique Seymour, Laura Pecora, Grace McMahon, Catherine E Wood, Mark Feinberg, Rob Hock and Rebecca Giallo in Autism
