# Supplementary material for: Trajectories of psychological distress for Australian fathers parenting a child on the autism spectrum: Evidence from early childhood to adolescence
Source: Autism. 2024 Sep 5;29(1):182–94. doi: 10.1177/13623613241272005 (PMC11656631; doi:10.1177/13623613241272005)
Supplement: sj-docx-1-aut-10.1177_13623613241272005 – Supplemental material for Trajectories of psychological distress for Australian fathers parenting a child on the autism spectrum: Evidence from early childhood to adolescence [file sj-docx-1-aut-10.1177_13623613241272005.docx]

Supplementary Table. Correlations between fathers’ psychological distress (K6) at each timepoint and potential predictor variables, pooled across 20 datasets.

|  | Psychological Distress (K6) | | | | | |
| --- | --- | --- | --- | --- | --- | --- |
|  | 4-5  years | 6-7  years | 8-9  years | 10-11 years | 12-13 years | 14-15 years |
| *Individual* |  |  |  |  |  |  |
| Father age | .010 | -.053 | .002 | -.063 | -.035 | -.090 |
| Father medical condition | **.167**** | .017 | **.125*** | .119 | .052 | -.031 |
| *Interpersonal* |  |  |  |  |  |  |
| Child sex | .028 | -.077 | -.025 | .061 | .071 | .125 |
| Child medical conditions | -.032 | -.064 | -.004 | -.042 | -.062 | -.136 |
| Child sleep problems | -.111 | -.065 | -.062 | .031 | -.031 | -.063 |
| P1 rated SDQ | **.174*** | **.149*** | .122 | **.231**** | .128 | .149 |
| Mothers’ K6 | **.167*** | **.195**** | .111 | .022 | .031 | .091 |
| Father Interparental conflict | **.321**** | **.219**** | **.256**** | **.143*** | **.246*** | .062 |
| Number of children in household | .035 | .066 | .055 | .058 | **.191*** | .093 |
| *Social environment* |  |  |  |  |  |  |
| Stressful life events | .093 | -.021 | .031 | -.033 | -.055 | -.093 |
| Education | .005 | .008 | .031 | -.032 | .021 | -.050 |
| Employment | -.057 | -.068 | -.055 | -.014 | -.024 | -.095 |
| ATSI | **-.164*** | -.087 | -.038 | -.148 | -.134 | -.004 |
| Country of birth | -.036 | -.035 | -.068 | -.066 | -.073 | **-.142*** |
| Remoteness | -.065 | -.025 | -.025 | .021 | .0256 | .004 |
| Financial hardship | .098 | .076 | **.167*** | .036 | .041 | -.046 |
| Job Quality | -.143 | -.146 | -.060 | -.120 | -.024 | -.119 |

** significant at p<.01; * significant at p<.05
